# *Enterobacter* Strain IPPBiotE33 Displays a Synergistic Effect with *Bacillus thuringiensis* Bt185

**DOI:** 10.3390/ijms241814193

**Published:** 2023-09-16

**Authors:** Liang Mi, Ziqiong Gu, Ying Li, Wenyue Xu, Changlong Shu, Jie Zhang, Xi Bai, Lili Geng

**Affiliations:** 1 State Key Laboratory for Biology of Plant Diseases and Insect Pests, Institute of Plant Protection, Chinese Academy of Agricultural Sciences, Beijing 100193, China; 2 College of Life Sciences, Northeast Agricultural University, Harbin 150038, China

**Keywords:** Enterobacter, *Bacillus thuringiensis*, coleopteran pests, *Holotrichia parallela*, synergistic effect

## Abstract

The discovery and isolation of new non-Bt insecticidal bacteria and genes are significant for the development of new biopesticides against coleopteran pests. In this study, we evaluated the insecticidal activity of non-Bt insecticidal bacteria, PPBiotE33, IPPBiotC41, IPPBiotA42 and IPPBiotC43, isolated from the peanut rhizosphere. All these strains showed insecticidal activity against first- and third-instar larvae of *Holotrichia parallela*, *Holotrichia oblita, Anomala corpulenta* and *Potosia brevitarsis*. IPPBiotE33 showed the highest toxicity among the four strains and exhibited virulence against *Colaphellus bowringi*. The genome of IPPBiotE33 was sequenced, and a new protein, 03673, with growth inhibition effects on *C. bowringi* was obtained. In addition, IPPBiotE33 had a synergistic effect with *Bacillus thuringiensis* Bt185 against *H. parallela* in bioassays and back-inoculation experiments with peanut seedlings. IPPBiotE33 induced a decrease in hemocytes and an increase in phenol oxidase activity in *H. parallela* hemolymph, known as the immunosuppressive effect, which mediated synergistic activity with Bt185. This study increased our knowledge of the new insecticidal strain IPPBiotE33 and shed new light on the research on new insecticidal coaction mechanisms and new blended pesticides.

## 1. Introduction

The order of coleopteran insects commonly known as beetles have hardened front wings, called elytra, as their defining feature. Scarabaeoidea and Chrysomeloidea are currently the most destructive coleopteran pests. White grubs, a major type of underground pest in Coleoptera, are considered to be a challenge in pest management worldwide [1,2]. They mainly damage corn, peanuts and soybeans, and their hazards are spread throughout multiple provinces in China. The yield loss is approximately 10~80% [3,4]. The white grubs that infest peanuts are mainly *Holotrichia parallela* Motschulsky, *Holotrichia oblita* Faldermann, and *Anomala corpulenta* Motschulsky, of which *H. parallela* is the most invasive [5,6]. *Colaphellus bowringi* Baly is a Chrysomelidae pest in Asia, especially in China. Larvae feed on the mesophyll, and adults mostly damage the leaves of cruciferous crops, including cabbage and radish [7]. Field infestations peak in spring and fall, with 1 generation in spring and 1–3 generations in autumn.

Currently, coleopteran pests are mainly controlled with chemical insecticides, such as diazinon, imidacloprid, chlorpyrifos, and carbaryl for white grubs and dipterex and sumicidin for *C. bowringi* larvae [8,9]. However, chemical insecticides remain controversial because of their negative environmental implications. To control coleopteran pests more sustainably, biopesticides have received attention from researchers for their safety and environmental friendliness. *Bacillus thuringiensis* (Bt) plays an important role in biocontrol measures and is considered to be the most widely used biopesticide in the world. Improvements in the research application of Bt and its insecticidal toxins have developed rapidly since they were first reported in 1902 [10]. Our laboratory reported that the Bt strain Bt185 exhibited toxicity against the larvae of *H. parallela* and successfully constructed the engineered Bt strain G033A with insecticidal activity against both lepidopteran and coleopteran pests in 2006 [2,11]. Since then, many insecticidal proteins from Bt with activity against coleopteran pests have been reported by researchers in succession, including Cry8-like (Cry8Ab, Cry8Ga1, Cry8Ha, Cry8-like, Cry8Ea1, Cry8Fa1, Cry8Ia, Cry8Na, Cry8Ca) and Vip (Vip1Ad1, Vip2Ag1) for white grubs and Cry1Ba, Cry1Ea, Cry3Aa, Cry7Ab for *C. bowringi* [12,13]. However, with the prolonged use of Bt biopesticides and Bt transgenic crops worldwide, the potential for insect pests to develop resistance is increased, and the effectiveness of Bt biological control may be reduced. The discovery and isolation of new non-Bt bacteria and insecticidal substances are becoming increasingly significant.

Currently, studies on non-Bt bacteria have mainly focused on Paenibacillaceae, Morganellaceae, Yersiniaceae and Pseudomonadaceae. *Paenibacillus popilliae* (Bacillales Paenibacillaceae) were reported as pathogens of *Popillia japonica* (Coleopteran Scarabaeidae) and *Papuana uninodis* (Coleopteran Scarabaeidae) [14,15]. Another Paenibacillaceae bacterium, *Brevibacillus laterosporus* (Bacillales Paenibacillaceae), was discovered to be toxic to *Plutella xylostella* (Lepidoptera Plutellidae) and to produce chitinases [16]. For strains in Morganellaceae, multiple insecticidal substances were reported, including Txp40, XaxAB, Tcs, Mcf, PVC and Pir [17,18,19,20,21,22]. Insecticidal research on Yersiniaceae and Pseudomonadaceae has usually focused on chitinases [23]. Chitinases extracted from *Pseudomonas fluorescens* (Pseudomonadales Pseudomonadaceae) MP-13 showed toxicity against *Helopeltis theivora* (Hemiptera Miridae) [24]. Chitinase *Pachi* from *Pseudomonas aeruginosa* (Pseudomonadales Pseudomonadaceae) was reported to enhance the insecticidal activity of Cry21Aa [25]. Other studies have reported that *Serratia proteamaculans* (Enterobacterales Yersiniaceae) AGR96X produced an anti-feeding prophage AfpX against white grubs [26]. Supernatants from cultures of *Serratia marcescens* were reported to enhance the larvicidal activity of the Bt toxin CrylC [27]. Recently, *Bacillus sphaericus* [28] and *Glutamicibacter halophytocola* [29] have also been reported to have the ability to synergize with Bt.

In our previous work, we found that a plant could recruit insecticidal bacteria to address the threats of insect pests and four non-Bt bacteria—IPPBiotE33 (*Enterobacter hormaechei*), IPPBiotC41 (*Citrobacter amalonaticus*), IPPBiotA42 (*Acinetobacter calcoaceticus*) and IPPBiotC43 (*Citrobacter amalonaticus*)—with high toxicity against *H. parallela* were obtained based on this phenomenon [30]. However, the insecticidal spectra of the four new strains and the insecticidal gene of IPPBiotE33 were unclear. The synergistic effect of IPPBiotE33 with Bt185 was also investigated to explore the prospective applications of non-Bt insecticidal bacteria. This study may shed new light on the development of new biopesticides.

## 2. Results

### 2.1. The Strains Showed Specific Toxicity against Coleopteran Insects

Insecticidal activities of four strains (IPPBiotE33, IPPBiotC41, IPPBiotA42, IPPBiotC43) against coleopteran and lepidopteran pests were assessed using oral and injection bioassays. The four strains showed greater activity against *P. breviaries* than against *H. oblita* and *A. corpulenta* (Table 1). *E. hormaechei* IPPBiotE33 showed the highest activities against white grubs among the four strains, and the LC_50_ values were 3.71 × 10^8^, 8.40 × 10^8^ and 0.19 × 10^8^ CFU/g for the first-instar larvae of *H. oblita*, *A. corpulenta* and *P. brevitarsis*, respectively (Table 1). The insecticidal activities against third-instar larvae of white grubs were also tested using injection bioassays. IPPBiotE33 also showed the highest activities among the four strains, and the corrected mortalities were 68.87%, 59.33%, 61.76% and 44.10% for *H. parallela*, *H. oblita, A. corpulenta* and *P. brevitarsis*, respectively (Table 2). In addition, it also exhibited particular toxicity against *C. bowringi* (LC_50_ value: 5.18 × 10^8^ CFU/g). For third-instar larvae, all four strains showed greater activity against *H. parallela* than against the other three grubs. However, their corrected mortalities against the lepidopteran insects *H. armigera* and *A. ypsilon* were less than 25% (Table 2). The above results indicated that the 4 strains isolated from *H. parallela* feeding peanut rhizosphere showed special insecticidal activity against Coleopteran insects but had no or less toxicity against Lepidopteran pests.

### 2.2. Analysis of the Whole Genome and Insecticidal Protein for IPPBiotE33

**Figure 1:**
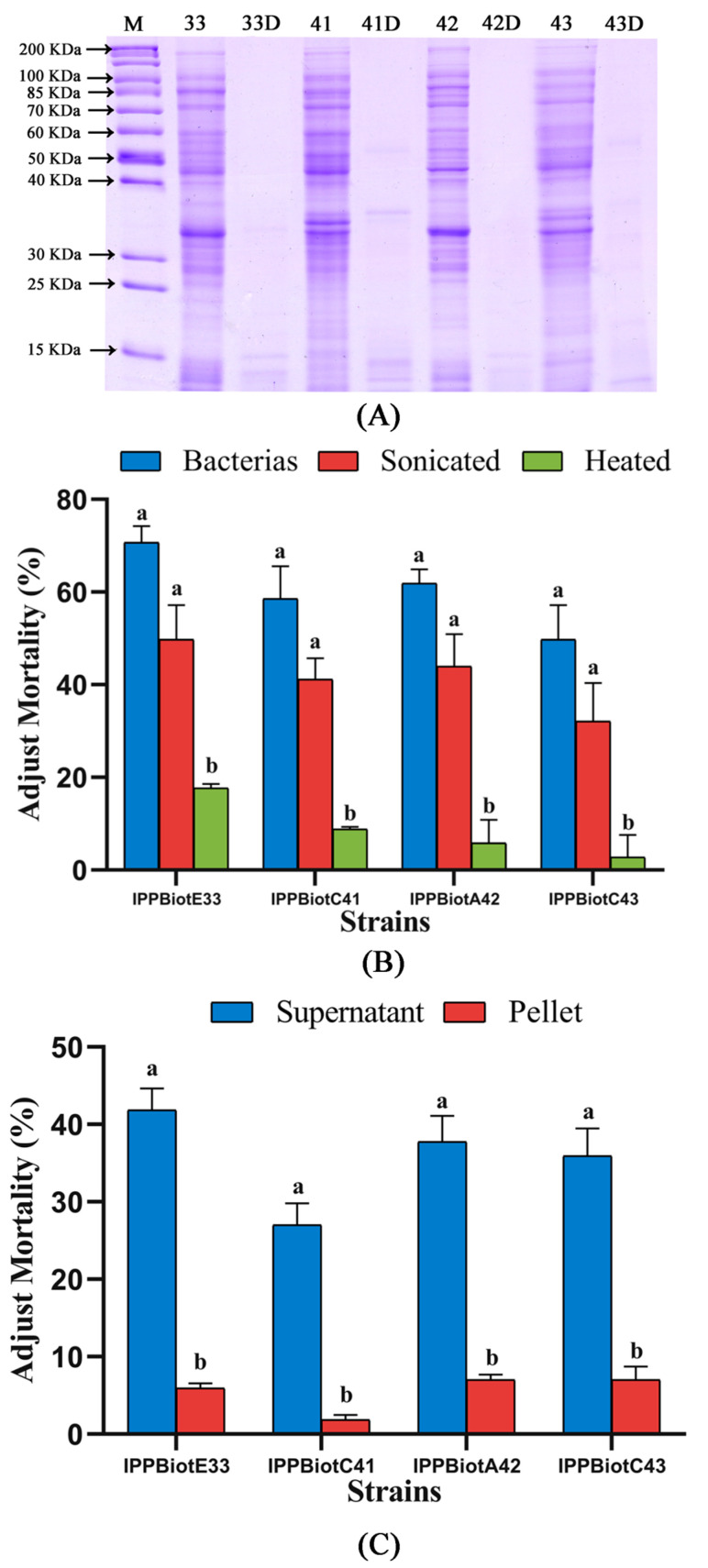
Mortality changes of denatured proteins compared with bacterial suspensions and sonicated suspensions. (**A**) SDS-PAGE was used to check the situation of extract protein disruption by 0.1 mg/mL Protease K. Lane M = marker. Lanes 33, 41, 42, 43 = proteins of IPPBiotE33, IPPBiotC41, IPPBiotA42 and IPPBiotC43. Lanes 33D, 41D, 42D, 43D = suspensions postprotease digestion. (**B**) Change in insecticidal activity of sonicated suspensions against *H. parallela* after heat and digestion (*n* = 12). (**C**) Insecticidal activity of centrifugation supernatant and precipitate of sonicated suspensions against *H. parallela* (*n* = 30). Bars with different letters denote significant differences at *p* < 0.05 determined by Student’s *t* test.

Heat denaturation (100 °C, 20 min) and protease digestion (0.1 mg/mL Protease K, 58 °C, 20 h) were used to disrupt the protein structure. SDS-PAGE showed that most of the proteins were almost denatured after protease digestion (Figure 1A). Denatured proteins were used for insect bioassays. The insecticidal activities of sonicated suspensions against *H. parallela* were substantially reduced after heat and digestion (Figure 1B,C). These results showed that proteins are the main sources of insecticidal activity for our strains.

**Figure 2:**
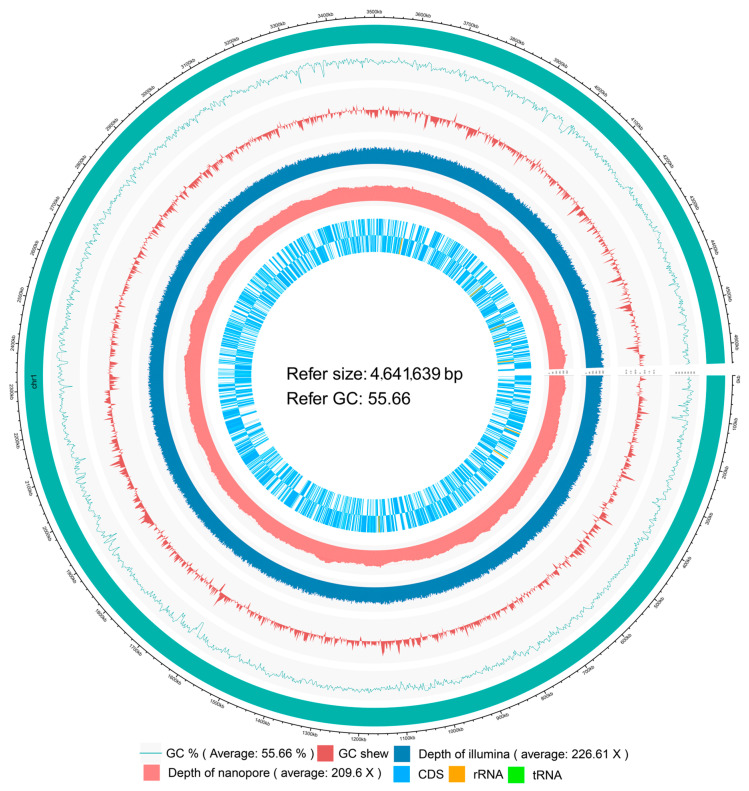
Circos plot of IPPBiotE33 (CP114033).

**Figure 3:**
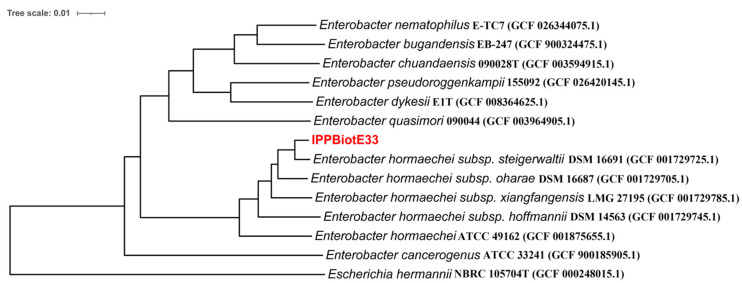
Phylogenetic tree based on the genome sequence for strain IPPBiotE33 and similar species on NCBI.

**Figure 4:**
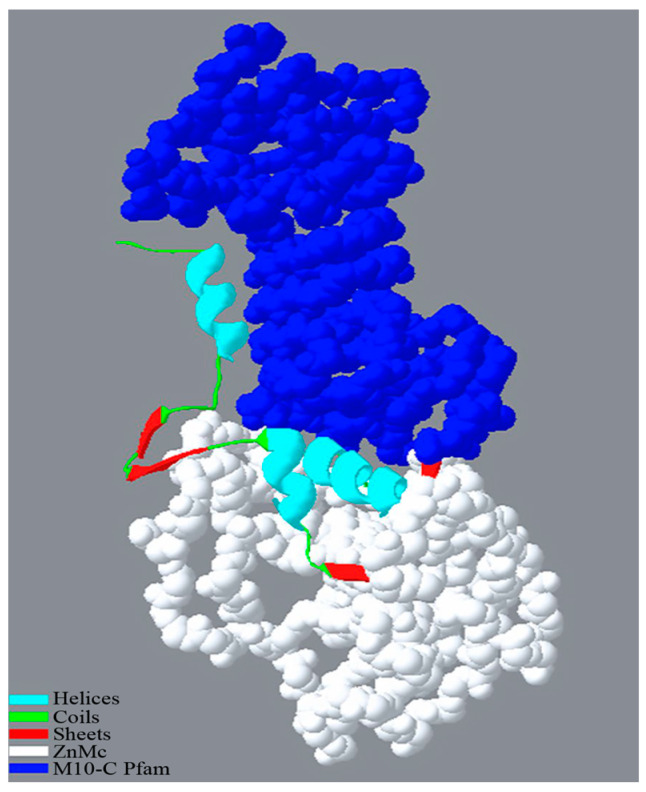
The 3D model of the protein 03673. Blue represents M10-C Pfam; white represents ZnMc.

**Figure 5:**
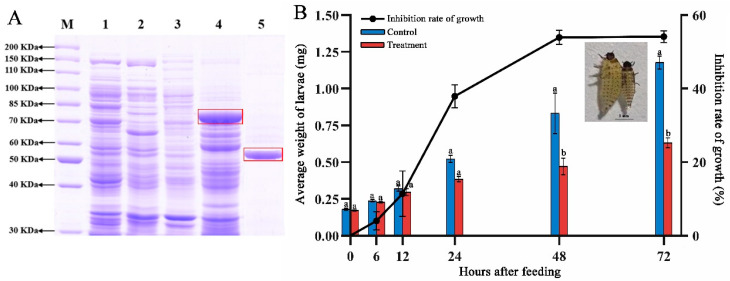
Purification and weight inhibition effect of protein 03673 on *C. bowringi*. (**A**) SDS-PAGE for purification of insecticidal protein 03673. Lane M = marker. Lane 1: ultrasound supernatant of BL21 (DE3) cells with pGEX4T1 empty vector. Lane 2: ultrasound pellet of BL21 with empty vector. Lane 3: ultrasound supernatant of BL21 with 03673-pGEX4T1 recombinant expression vectors. Lane 4: ultrasound pellet of BL21 with 03673-pGEX4T1. Lane 5: protein 03673 purified after denaturation and renaturation from the inclusion body. The target protein is framed. (**B**) Changes in the weight and inhibition rate of larvae. The histogram shows the weight change with time after feeding (n = 30 for each group). Blue represents control (feed PBS buffer) and red represents treatment (feed 0.2 mg/mL of protein 03673). Different letters denote significant differences at the same timepoint (*p* < 0.05) determined by Student’s *t* test. The line chart shows the inhibition of weight (n = 30 for each group). The picture shows the large shape change of *C. bowringi*. The larva on the left was fed PBS buffer, and the larva on the right was fed protein 03673.

The genome of IPPBiotE33 was sequenced, and 1,126,526,100 bp bases and 7,510,174 reads were obtained. After assembly, two contigs were combined. The chromosome is 4,641,639 bp in length (GenBank: CP114033, Figure 2) and the plasmid is 59, 140 bp in length (GenBank: CP114034). They are identical to their contig N50. The average sequencing depth is approximately 209.6X. In total, 4562 genes and 4305 CDSs were annotated (Table 3). A phylogenetic tree based on the whole-genome sequence was constructed, and the strain IPPBiotE33 is highly similar to the strain *Enterobacter hormaechei* subsp. *steigerwaltii* DSM 16691 (GenBank: CP077392.1) (Figure 3). The Average Nucleotide Identity between them is 98.97% by comparing their genomes. Therefore, the strain IPPBiotE33 was identified as *Enterobacter hormaechei*. We identified the functions of genes using BLAST+ with COG, KEGG, and GO and found that the metalloprotease gene *CDS-03673* (1494 bp, GenBank: OP846099) shared 76.63% similarity with *Pr596* from *Serratia marcescens* HR-3 (EF070725.1), which was reported as an insecticidal protein gene against locusts [31]. The isoelectric point and molecular weight of protein 03673 were predicted to be 4.26 and 53.26 kDa, respectively. It contains a zinc-dependent metalloprotease and a peptidase-M10-C Pfam domain (Figure 4), which could mean that 03673 is a matrix metallopeptidase (MMP) with zinc-binding activity. The *CDS-03673* gene was expressed in *E. coli* BL21 (DE3) cells. SDS-PAGE shows that the protein was present in the precipitate in the form of inclusion bodies. The inclusion bodies were purified, denatured and renatured and purified at approximately 53 kDa (Figure 5A). Protein 03673 at 0.2 mg/mL was used for the bioassays and demonstrated weight inhibition in *C. bowringi* larvae (Figure 5B). The weight of the treatment showed differences in the control from 6 h after feeding and showed significance from 48 h. The inhibition rate of weight grew continuously after 6 h and reached a plateau of 53.90% at 48 h.

### 2.3. The Synergistic Effect of IPPBiotE33 and Bt185

*Bacillus thuringiensis* Bt185 was explored in our laboratory and showed high toxicity against *H. parallela* larvae [11]. The synergistic effect of IPPBiotE33 and Bt185 was analyzed using the Wadley method. The individual LC_50_ values of strains IPPBiotE33 and Bt185 were 8.81 and 6.59 × 10^7^ CFU/g, respectively, while their combined LC_50_ value was 2.06 × 10^7^ CFU/g. The synergistic ratio (SR) of IPPBiotE33 and Bt185 was 3.66, which suggested that these two strains had a synergistic effect at a CFU ratio of 1:1 (Table 4).

**Figure 6:**
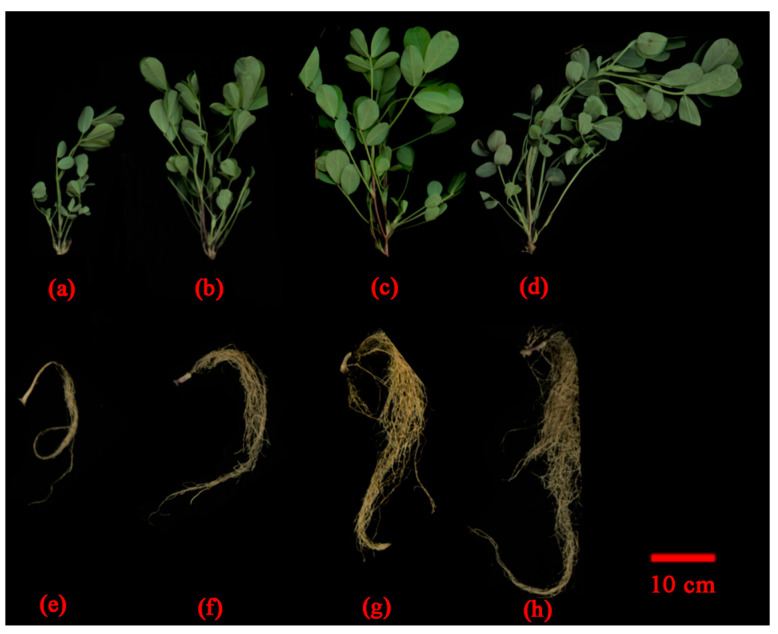
Changes to peanut plants after application of IPPBiotE33 and Bt185. (**a**–**d**) Show shoots of peanuts in treatment group PBS Buffer, IPPBiotE33, Bt185 and IPPBiotE33 + Bt185 on the 7th day. (**e**–**h**) Show roots of peanuts in treatment group PBS Buffer, IPPBiotE33, Bt185 and IPPBiotE33 + Bt185 on the 7th day.

A pot experiment was used to further check the synergistic effect of IPPBiotE33 and Bt185. We mixed bacterial suspensions of IPPBiotE33, Bt185 and their 1:1 mixture at a concentration of 10^9^ CFU/g into the soil and planted peanut seedlings. Seven days later, two 2-day-old *H. parallela* larvae were inoculated near the roots of each peanut seedling. The changes in root length, shoot height, fresh weight and dry weight of peanuts were observed after seven days. The four physiological indicators of the seedlings for IPPBiotE33, Bt185 and the 1:1 mixture were higher than the control (PBS buffer) (Table 5, Figure 6). This demonstrated that the three bacterial suspensions enhanced peanut resistance to *H. parallela* larvae. In addition, the shoot height and the fresh weight of the IPPBiotE33 and Bt185 mixture were greater than those of the single bacterial suspension. The weight loss rate for the mixture was also higher than that for the single component (Table 5). The application of IPPBiotE33 and Bt185 to peanut seedlings also showed a synergistic effect against *H. parallela* larval feeding.

### 2.4. Immunosuppressive Activity of IPPBiotE33 in H. parallela

**Figure 7:**
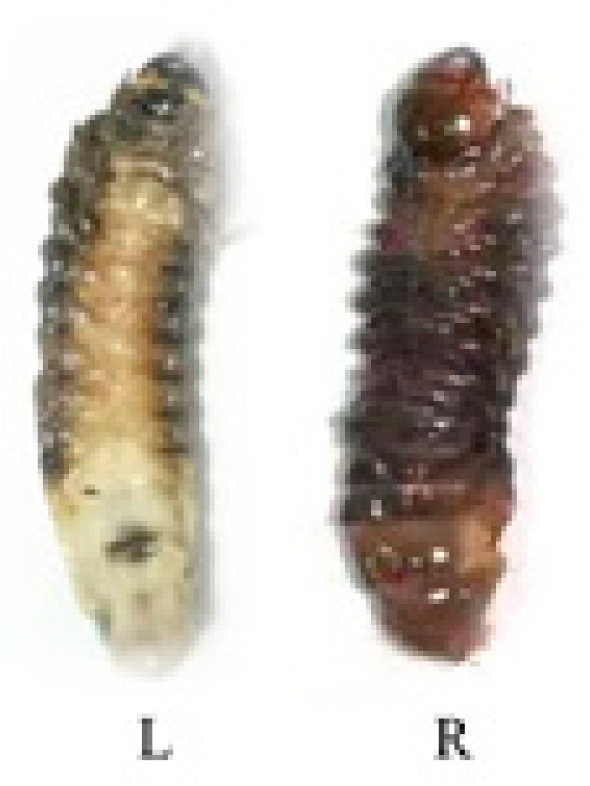
Melanization reaction and hemolymph discoloration in IPPBiotE33-challenged larvae at 24 h after injection of 5 × 10^5^ CFU/larva. (L) Larva injected with PBS. (R) Larva injected with 5 × 10^5^ CFU/larva IPPBiotE33 bacterial suspension.

**Figure 8:**
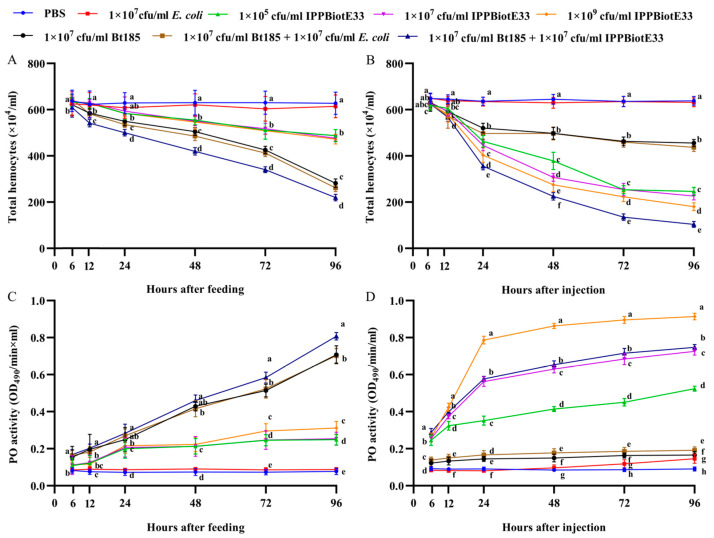
Effects of IPPBiotE33 and Bt185 on hemocyte concentration and PO activity of *H. parallela*. (**A**,**B**) show changes in hemocyte concentration through 96 h post feeding or injection. The *y*-axis indicates total hemocyte counts (×10^4^) per milliliter in each treatment. (**C**,**D**) show changes in PO activity in hemolymph through 96 h post feeding or injection. Insects fed or injected with PBS or *E. coli* were used as the control. All values are represented by the mean ± standard error (*n* = 18). Bars with different lowercase letters denote significant differences at the same time points at *p* < 0.01 determined by Tukey’s multiple comparisons.

Larvae exhibited a melanization reaction and hemolymph discoloration after the IPPBiotE33 experiment (Figure 7). This suggests that IPPBiotE33 may exert its function in the hemocoel. Hemocytes often decrease when insects are challenged with immunosuppressive molecules [32]. Abiotic and biotic stressors can cause insects to increase melanization as a result of elevated phenol oxidase (PO) levels in their hemolymphs due to the conversion of hemocyte-bound pro-phenol-oxidase (proPO) to PO [33]. We determined the hemocyte concentration and PO activity of *H. parallela* post feeding or injection. The hemocyte concentration of larvae fed IPPBiotE33 decreased significantly with prolonged time in a dose-independent manner compared with the blank control (*E. coli*) and negative control (PBS) (Figure 8A). The number of hemocytes decreased even more after feeding on Bt185 or Bt185 and *E. coli* than after feeding on IPPBiotE33. The most drastic reduction in hemocyte concentration was the treatment of feeding both IPPBiotE33 and Bt185. For injection treatments, the declines in hemocyte numbers caused by Bt185 or Bt185 and *E. coli* were fewer than those caused by different doses of IPPBiotE33 (Figure 8B). As the concentration of IPPBiotE33 increased, the number of hemocytes decreased even more. Similar to the feeding treatment, injection of both IPPBiotE33 and Bt185 resulted in the greatest reduction in hemocyte concentration.

The PO activity was also analyzed. In the feeding treatment, IPPBiotE33 increased the PO activity in a concentration-dependent manner such that 10^9^ CFU per larva caused a higher PO value at 72 h and 96 h (Figure 8C). Bt185 or Bt185 and *E. coli* induced a greater increase in PO activity than feeding on IPPBiotE33. Feeding with both IPPBiotE33 and Bt185 caused the highest PO activity. For injection treatment, Bt185 or Bt185 and *E. coli* had only minor effects on PO activity (Figure 8D). IPPBiotE33 induced dose-dependent effects on PO activity, and 10^9^ CFU per larva showed the most dramatic increases. Injection of both IPPBiotE33 and Bt185 caused a greater effect on the PO value compared with IPPBiotE33 (10^7^ CFU/larva) or Bt185 (10^7^ CFU/larva) alone. The results indicated that IPPBiotE33 could induce an immunosuppressive effect on *H. parallela* hemocytes, which mediated the synergistic effect with Bt185.

## 3. Discussion

Highly effective control of underground coleopteran pests, especially white grubs, is a major puzzle in agriculture. As the most useful microbe for controlling insects worldwide, Bt is also the most-cited bacterium that kills white grubs. The Bt185 strain showed toxicity against *H. parallela* larvae with an LC_50_ value of 0.9464 × 10^8^ (CFU/mL) [11]. Bt HBF-1 showed an LC_50_ of 34.713 μg/g soil for *A. corpulenta* [34]. The strain HBF-18 even showed an LC_50_ of 0.2043 μg/g soil for *H. oblita* and 0.1078 μg/g soil for *H. parallela* [35]. Some non-Bt grub-killing bacteria were also reported. *Paenibacillus popilliae* and *Paenibacillus lentimorbus* are a signature combination of non-Bt insecticidal bacteria that showed high toxicity against *Popillla japonica* (Coleopteran Scarabaeidae) and were widely used in the 1940s in an American control program for white grubs [36]. However, even after several reformulations, they continue to show low levels of infectivity and efficacy in field trials [37]. *Salmonella haemophilus* was the causal agent of Amber Disease in New Zealand grass grubs (*Costelytra zealandica*) and developed as a commercial product in New Zealand in the 1980s [38]. It can increase the incidence of amber disease in grass grub populations by 20% and is still available in a liquid formulation [39]. In addition, some other non-Bt bacteria were found to be effective against grubs but are not currently in widespread use. The *Serratia proteamaculans* strain AGR96X showed an LD_50_ of 5 × 10^3^ CFU per larvae against grass grubs in 12 days [26]. A *Yersinia entomophaga* isolated from grass grubs reduced the number of adult grubs by half at 70 kg ha^−1^ [40,41]. In China, the *Bacillus megaterium* strain ZLP-21 was reported to be 71.11% lethal to *Potosia brevitarsis* when fermented for 48 h and diluted tenfold [42].

**Figure 9:**
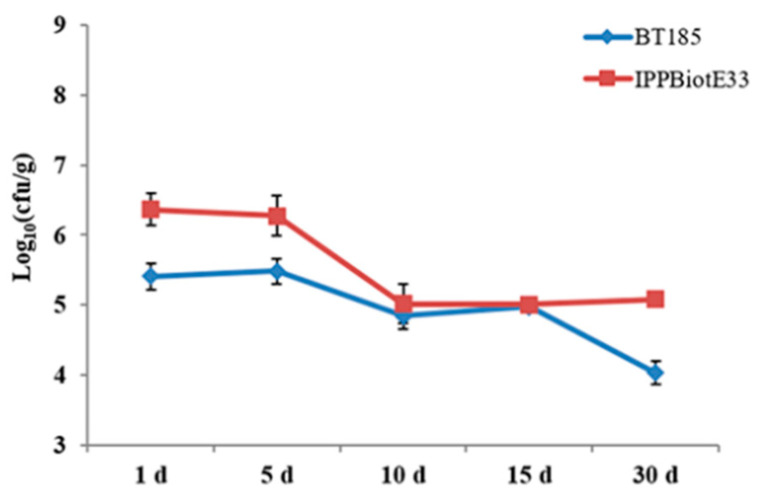
Dynamic changes in bacteria Bt185 and IPPBiotE33 colonizing peanut roots. The *x*-axis indicates days after irrigation, and the *y*-axis indicates abundance shifts of bacteria.

The four new bacteria we mentioned in this study (IPPBiotE33, IPPBiotC41, IPPBiotA42, IPPBiotC43) may shed light on the development of biopesticides. They are similar in activity to the Bt we have often used in white grub control (Bt185, HBF-1, HBF-18) and are more effective than most of the reported non-Bt grub killers. We found that many of the grub-killing bacteria belonged to the order Enterobacterales, including IPPBiotE33, IPPBiotC41 and IPPBiotC43. This indicated that strains belonging to the order Enterobacterales had a certain advantage in the control of white grubs. Normally, bacteria of the genus *Enterobacter* are more abundant in soil, in the inter-rhizosphere of plants and in the gut of insects [43]. These characteristics are also reflected in IPPBiotE33. According to our experiments, IPPBiotE33 shows a good colonization capacity (Figure 9). In terms of pathogenesis, feeding on IPPBiotE33 will cause immunosuppression in insect hemolymph. This means that IPPBiotE33 is able to survive in the insect’s gut and possibly cause insect septicemia. This suggests that IPPBiotE33 has the potential to be developed into a new type of biopesticide for the control of coleopteran pests.

In terms of insecticidal substances, most of the insecticidal proteins currently effective against coleopteran pests are derived from Bt, including Cry1, Cry3, Cry7, Cry8, APP6, Cyt1, MPP5 and Vip1/Vip2 [12,44,45]. For instance, Cry8Ha1 and Cry8Ia1, as prototypical Cry8-like proteins used in controlling coleopteran pests, exhibit LC_50_ values of 21.89 and 8.50 (10^9^ CFU/g), respectively, against *H. parallela* larvae. In contrast, insecticidal proteins derived from bacteria belonging to the *Enterobacteriaceae* family are extremely rare. It was not until 2019 that Morishita et al. reported the initial detection of a toxin complex (Tc) against *Bombyx mori* silkworms produced by *Enterobacter* sp. strain 532. Nonetheless, the level of its activity was not specified in the report [46,47]. The metalloprotease protein 03673, which we discovered from *Enterobacter* sp. strain IPPBiotE33, exhibited inhibitory effects on the larval development of *C. bowringi*. This discovery is expected to enhance and enrich research in this field.

Metalloproteases are often of research interest due to their hydrolytic catalytic activity and their applications in food processing [48]. Several studies exploring the insecticidal activity of metalloproteases have been published to date. In 2017, Lee et al. reported that AprA, an alkaline zinc metalloprotease from the genus *Pseudomonas*, is an important insecticidal protein against *Riptortus pedestris* through the suppression of host cellular and humoral innate immune responses [49]. A half year later, Aydin et al. reported that baculoviruses containing iridoviral metalloproteases caused early mortality in *Galleria mellonella* larvae [50]. Diab et al. reported the discovery of a 27.33 kDa astacin-like metalloprotease in 2021 that demonstrated mortality ratios of 69.3%, 65% and 64.0% against first-instar *Spodoptera littoralis*, second-instar *S. littoralis* and adult *Sitophilus oryzae*, respectively, at a concentration of 1000 μg/mL [51]. Our research on the metalloprotease protein 03673 provides a new example in this area and may raise questions about the insecticidal effects of metalloproteases.

Promoting the effectiveness of Bt formulations has been a hot topic since their application. Thus far, a wide variety of Bt synergists have been identified, including both insecticidal and non-insecticidal compounds. Chitinase has been reported to possess both insecticidal and antifungal activities and has been reported to decrease the LC_50_ of Bt protein against *Spodoptera litura* larvae by 30% [52]. Regarding non-insecticidal compounds, research on zwittermicin A has been a significant area of interest in the advancement of Bt synergists. The addition of 2.5 ng/disk zwittermicin A was reported to increase the average Bt mortality of gypsy moth larvae from 25% to 100% [53]. Wang et al. also reported that the extracellular polysaccharides of Bt 4D19 increased the activity of its total protein against *Plutella xylostella* by 15% [54]. Our research demonstrated synergistic insecticidal activity between IPPBiotE33 pairs of Bt185, providing a foundation for the discovery of new Bt synergists. Unexpectedly, a synergistic effect persisted in the hemolymph analysis of our results, indicating the existence of potentially interesting interactive mechanisms between them that warrant further investigation. In this respect, we have two hypotheses. On the one hand, Bt can cause midgut perforation to allow IPPBiotE33 to enter the hemolymph and induce lesions. On the other hand, the secretions of IPPBiotE33 may make Bt toxins more active. We speculate that both mechanisms may exist simultaneously, and this could be tested in future experiments.

In our findings, the performance of Bt in immunoassays is noteworthy. Bt185 did not demonstrate independent immunosuppressive activity in the bloodstream. This suggests that the mortality of the larvae is not dependent on septicemia caused by Bt. There is ongoing debate regarding the pathogenesis of Bt. Some scholars believe that Bt alone can cause insect death, while other bacteria in the gut have a negative effect or no effect at all. In a 2009 study, Raymond et al. found that the presence of gut microbiota reduced the pathogenicity of the Bt toxin Cry1Ac. Therefore, they suggested that the midgut microbiota is not required for the pathogenicity of Bt against diamondback moth larvae [55]. Buisson et al. suggested that Bt spores could act independently in the *G. mellonella* hemocoel [56]. Clearly, these findings are contradictory to the results of our research. Other scholars found that Bt alone is insufficient to eliminate insects and that gut microbiota are crucial in facilitating the insecticidal properties of Bt, which was consistent with our results. When *Manduca sexta* larvae were fed Bt toxin alone, microbiota-free larvae died of starvation, while when Bt toxin was ingested along with *Enterococcus faecalis*, the larvae died of acute infection [57]. Caccia and Li previously published articles describing the importance of the gut microbiota in the pathogenicity of Bt in 2016 and 2022, respectively [58,59]. Our results and the above reports indicated that the lethal effect of Bt might be the combined action of multiple pathways and that the microorganisms in the gut might be involved, but this is not absolute and is related to the species of insects and microorganisms. In conclusion, although our research has enriched the basis for addressing this issue, definitive evidence in this area is still lacking, and future research is expected.

## 4. Materials and Methods

### 4.1. Bacterial Strains and Growth Conditions

Bacterial strains IPPBiotE33, IPPBiotC41, IPPBiotA42 and IPPBiotC43 were originally separated from rhizosphere soil samples of peanuts and showed high toxicity against first-instar larvae of *H. parallela* in our previous work. IPPBiotE33 (CGMCC No. 20449) and IPPBiotC41 (CGMCC No. 22672) were deposited at the CGMCC Center and IPPBiotA42 and IPPBiotC43 were maintained in our laboratory. *B. thuringiensis* Bt185 was also toxic to *H. parallela* larvae and maintained in our laboratory. Luria–Bertani (LB) broth was used for growing all strains at 30 °C. The phylogenetic tree for strain IPPBiotE33 was based on the genome sequence of this strain and its similar species. The genome sequences were obtained from NCBI “https://www.ncbi.nlm.nih.gov (accessed on 15 July 2023)” and the phylogenetic tree was constructed using the website “https://ggdc.dsmz.de/ggdc.php# (accessed on 3 July 2023)”.

### 4.2. Insect Rearing and Bioassays

*Agrotis ypsilon* Rottemberg (Lepidoptera: Noctuidae) and white grubs, including *H. parallela*, *H. oblita*, *A. corpulenta* and *Potosia brevitarsis* Lewis, were provided by the Institute of Plant Protection, CangZhou Academy of Agriculture and Forestry Sciences, CangZhou, China. *C. bowringi* and *Helicoverpa armigera* Hübner (Lepidoptera: Noctuidae) larvae were mass-reared from eggs in our laboratory. All insects were reared at 25 °C with a relative humidity of 50%. *A. ypsilon* and *H. armigera* were reared in 12 h light:12 h dark; *C. bowringi* in 16 h light:8 h dark; and white grubs in darkness.

The leaf dipping method according to Gao was used for the bioassay of insects [60]. Chinese cabbage leaves were soaked in sample suspensions containing 0.1% surfactant L-77 (Solarbio Science & Technology Co., Ltd., Beijing, China) for 5 min and air dried in Petri dishes. Thirty first-instar larvae were inoculated on each Chinese cabbage leaf, and each treatment was repeated 3 times. Mortality rates were scored after 48 h.

Bioassays of *A. ypsilon* and *H. armigera* used artificial feed and followed the method described by Shankhu [61]. Two milliliter samples with 20 g feed were mixed and air dried in Petri dishes. Thirty first-instar larvae of *A. ypsilon* were inoculated in each Petri dish. For *H. armigera*, the feed was evenly distributed in a 24-well culture plate, and 1 larva was inoculated into each well. Each treatment was repeated 3 times. Mortality rates were calculated after 7 d.

Insect bioassays for larvae of white grubs were conducted following the method described by Yu et al. with some modifications [2]. Carrots were cut into shreds, washed and dried. Carrot shreds were soaked in 20 mL of bacterial suspension for 20 min, and the remaining suspension was evenly mixed in 90 g of soil (humidity of 18–20%). Carrot shreds were evenly distributed into three 6-well culture plates and covered with soil. Each well was inoculated with 2 newly hatched first-instar larvae, and each treatment was repeated 3 times. Larval mortality was scored after 7 days.

Third-instar larvae of white grubs were used for injection bioassays. Larvae of white grubs were surface sterilized with cotton swabs dipped in 70% ethanol. Sterilized syringes were used to inject 50 μL of bacterial suspension into each larva. The inoculated larvae were kept in 50 mL plastic tube filled with soil (humidity of 18–20%), one larva in each tube. Larval mortality was scored after 7 days.

In all insect bioassay experiments, death was determined by poking and prodding the larvae and observing their condition and response, and mortality rates were used to describe the efficacy.

### 4.3. Genomic DNA Extraction and Sequencing

Total genomic DNA was extracted using the CTAB method [62]. Whole-genome sequencing was completed by Wuhan Benagen Technology Co., Ltd, Wuhan, China. A Unicycler (Version: 0.5.0) was used to assemble combined Illumina and nanopore sequencing reads [63]. To create Circos plots, we used the R package circlize [64]. To predict genes on newly assembled contigs, Prokka (Version: 1.14.6) was used [65]. Gene functions were identified using BLAST+ (Version: 2.11.0+) “https://blast.ncbi.nlm.nih.gov/Blast.cgi (accessed on 13 January 2022)” with COG, KEGG and GO [66,67,68]. The protein domains were identified and annotated using SMART “https://smart.embl.de (accessed on 13 January 2022)” [69]. The 3D model of the protein was made usingS SPDBV (Version: 4.1.0, Version: 2.11.0+).

### 4.4. Discovery and Purification of Insecticidal Protein 03673

A 300 mL culture of bacteria was grown in Luria–Bertani (LB) broth at 37 °C until the OD_600_ reached 0.6. Bacterial pellets obtained from centrifugation (6000 rpm, 10 min) were resuspended in PBS buffer and lysed via sonication to extract crude protein. Heat denaturation (100 °C, 20 min) and protease digestion (0.1 mg/mL Protease K, 58 °C, 20 h) were used to disrupt the protein structure. The primers 5′-CCGGAATTCATGTTTATCTATTTGATCGTT-3′ (forward primer), with an *EcoRI* site (underlined), and 5′-CCGCTCGAGGACGATAAAGTCGGTGGCGAC-3′ (reverse primer), with an *XhoI* site (underlined), were synthesized based on the sequences of *33-03673* for amplification of the full-length gene. The PCRs were carried out using PrimerSTAR Max DNA polymerase (TaKaRa, Beijing, China) to amplify the gene. The cycling conditions for the PCRs were as follows: 98 °C for 10 min, 30 cycles of denaturation at 98 °C for 10 s, annealing at 56 °C for 2 min, and extension at 72 °C for 1 min, followed by a 10 min extension at 72 °C. The amplified *33-03673* gene was ligated into the pGEX4T1 vector and transformed into *E. coli* BL21 (DE3) cells.

The transformed colony was grown in LB broth at 37 °C. When the OD_600_ of the bacterial culture reached 0.6, 1 mM isopropyl-β-D-thiogalactopyranoside (IPTG) was used for recombinant protein induction at 16 °C for 12 h. The pellet was obtained via centrifugation (6000 rpm, 10 min) and suspended in 50 mM Tris-HCl buffer. The suspension was lysed via sonication and checked by 10% SDS-PAGE. The serralysin-like protein 33-03673 was purified via denaturation and renaturation from the inclusion body using the Inclusion Body Protein Extraction Kit (Sangon, Shanghai, China) and tested by SDS-PAGE.

### 4.5. Synergistic Analysis

The synergism ratio (SR) was a measure of whether there was synergism between the two solutions and the Wadley method was used to calculate it [70]. The Lethal Concentration 50 (LC_50_) value in the following equation was calculated using SPSS (version 19.0) with reference to Finney’s method [71].

$$\mathrm{SR}=\frac{{\mathrm{LC}}_{50\exp}}{{\mathrm{LC}}_{50\mathrm{obs}}}=\frac{P_{A}{+P}_{B}}{{\mathrm{LC}}_{50\mathrm{obs}}\times \left(\frac{P_{A}}{{\mathrm{LC}}_{50A}}+\frac{P_{B}}{{\mathrm{LC}}_{50B}}\right)}$$

LC_50exp_ is the expected LC_50,_ and LC_50obs_ is the observed LC_50_ of the mixing solution. P_A_ and P_B_ are the percentages of solutions A and B in the mix. LC_50A_ and LC_50B_ are the LC_50_ of single solution A or B. SR ≥ 1.5 indicates the synergistic effect, 1.5 > SR > 0.5 is a summation effect, and SR ≤ 0.5 is an antagonistic effect.

### 4.6. Pot Experiment and the Evaluation of Colonization Abilities

Peanut seedlings were grown in pots containing turfy soil and a vermiculite mixture (volume ratio 2:1) mixed with IPPBiotE33 or Bt185 or their mixture at a concentration of 10^9^ CFU/g to investigate the effect of these bacterial fluids on the plants. PBS buffer was used as a negative control and 30 seedlings were established for each treatment. Seven days after germination, root and shoot lengths, and fresh and dry weights of seedlings were measured as initial values, and then two 2-day-old *H. parallela* larvae were inoculated near their roots after weighing. Seven days after larval inoculation, larvae were collected from the soil and the condition of the plants was observed. The root and shoot lengths, the fresh and dry weights of the seedlings and the weights of the larvae at this time were recorded and their changes were calculated by subtracting the initial values.

### 4.7. Estimation of Hemocyte Concentration

Third-instar larvae of *H. parallela* were injected with 10 μL of bacterial suspension via the intersegmental membrane of the hemocoel using a sterilized syringe. The number of hemocytes was observed at 12 h after the injection of bacterial suspensions. Sterile needles were used to puncture the *H. parallela* larval cuticle dorsally, and 10 μL of hemolymph was extracted using a micropipette [72]. To differentiate dead and live hemocytes, the appropriate amount of 0.4% Trypan blue with anticoagulant was added to the extracted hemolymph. For each sample, the actual number of live hemocytes per milliliter was counted in a hemocytometer under the microscope.

### 4.8. Phenol Oxidase Activity in Insect Hemolymph

At 6 h postinjection, the phenol oxidase (PO) activity of *H. parallela* was assayed. Hemolymph (10 μL) was extracted from the larvae as described above. PO activity was measured as the formation of dopachrome from the substrate L-dihydroxyphenylalanine (L-DOPA) in 96-well microtiter plates [73]. Hemolymph samples with 100 μL of L-DOPA (4 mg/mL) were incubated for 30 min at 25 °C. A spectrophotometer was used to continuously measure the optical density at 490 nm absorbance for 10 min. One unit of PO activity was expressed as the increase in absorbance at OD_490_ per minute per milligram of protein. The OD_490_ value of the blank sample was subtracted from each sample.

### 4.9. Statistical Analysis

Data were checked for normality via the Kolmogorov–Smirnov test and compared using one-way ANOVA followed by SPSS (version 26.0.0.0). LC_50_ values and confidential limits were calculated using SPSS (version 19.0) according to Finney’s probit analysis LC_50_ determination method [71].

## 5. Conclusions

In this study, the insecticidal capacities of four new insecticidal strains (IPPBiotE33, IPPBiotC41, IPPBiotA42, IPPBiotC43) identified in our previous work were analyzed. The whole genome of IPPBiotE33 was sequenced, and a novel protein, 03673, that inhibited the growth of *C. bowringi* was obtained. We also found the synergistic effect of IPPBiotE33 with Bt185 and the immunosuppressive activity of IPPBiotE33 in *H. parallela*. These discoveries deepened our knowledge of the four new insecticidal strains and shed new light on the development of new pesticides and research into new insecticidal coaction mechanisms. IPPBiotE33, as one of them, has high potential for research and application due to its high colonization ability and synergistic effect with Bt185.

## Figures and Tables

**Table 1:** Bioassay analysis of IPPBiotE33, IPPBiotC41, IPPBiotA42 and IPPBiotC43 against first-instar larvae of *C. bowringi*, *H. oblita*, *A. corpulenta* and *P. brevitarsis*.

Strains	The LC_50_ Values (×10^8^ CFU/g) (95% Confidence Interval)			
	*C. bowringi*	*H. oblita*	*A. corpulenta*	*P. brevitarsis*
IPPBiotE33	5.18 (1.04–19.80)	3.71 (1.23–7.73)	8.40 (2.12–18.78)	0.19 (0.02–0.53)
IPPBiotC41	—	83.31 (25.33–191.62)	18.12 (2.86–52.96)	0.78 (0.12–1.89)
IPPBiotA42	—	351.16 (113.52–919.17)	10.43 (1.97–23.43)	1.16 (0.27–2.87)
IPPBiotC43	—	416.28 (114.84–911.42)	29.64 (2.99–85.74)	1.82 (0.30–4.41)

— The strain showed no toxicity against *C. bowringi*.

**Table 2:** The corrected mortality for strains injected or orally administered to third-instar grubs or first-instar *H. armigera* and *A. ypsilon*.

Strains	Corrected Mortality (Mean ± SD) (%)					
	Third-Instar Larvae				First-Instar Larvae	
	*H. parallela* ^a^	*H. oblita* ^a^	*A. corpulenta* ^a^	*P. brevitarsis* ^a^	*H. armigera* ^ab^	*A. ypsilon* ^b^
IPPBiotE33	68.87 ± 3.72	59.33 ± 2.01	61.76 ± 3.27	44.10 ± 3.57	21.91 ± 3.32	13.10 ± 2.72
IPPBiotC41	48.50 ± 1.70	34.57 ± 4.13	35.29 ± 2.51	28.43 ± 4.31	13.90 ± 9.01	11.90 ± 2.42
IPPBiotA42	54.33 ± 4.15	21.60 ± 4.10	50.00 ± 4.21	20.57 ± 3.70	14.60 ± 12.52	4.76 ± 1.14
IPPBiotC43	42.60 ± 1.51	23.53 ± 2.60	40.12 ± 1.94	19.63 ± 2.65	22.14 ± 7.12	10.71 ± 4.71

^a^ Each third-instar larva was injected with 10^8^ CFU for injection bioassays. ^b^ First-instar larvae were fed bacteria at a concentration of 10^8^ CFU/mL for oral bioassays.

**Table 3:** Basic genome information of IPPBiotE33.

Genomic Contents	Number
Plasmid	1
Gene	4562
CDS	4305
tRNA	85
rRNA	25

**Table 4:** Synergistic effect of IPPBiotE33 and Bt185 in a 1:1 mixture against first-instar *H. parallela*.

Strains	LC_50_ Values (10^7^ CFU/g) (95% Confidence Interval)	Synergic Ratio (SR)
IPPBiotE33	8.81 (1.53–22.40)	3.66
Bt185	6.59 (1.44–14.14)	
IPPBiotE33:Bt185 = 1:1	2.06 (0.38–4.63)	

**Table 5:** The effects of IPPBiotE33 and Bt185 on peanut plants and *H. parallela* larvae.

Data (Mean ± SD)	PBS Buffer	IPPBiotE33	Bt185	IPPBiotE33 + Bt185
Changes in root length (cm)	0.878 ± 0.630 ^b^	3.38 ± 0.597 ^a^	4.307 ± 0.519 ^a^	5.577 ± 0.814 ^a^
Changes in shoot height (cm)	0.971 ± 0.667 ^d^	3.961 ± 1.285 ^c^	4.096 ± 1.910 ^b^	4.265 ± 2.180 ^a^
Changes in fresh weight (g)	1.518 ± 0.305 ^c^	4.105 ± 0.351 ^b^	5.041 ± 1.948 ^b^	8.486 ± 1.710 ^a^
Changes in dry weight (g)	0.502 ± 0.519 ^c^	1.387 ± 0.490 ^b^	1.484 ± 1.375 ^ab^	2.063 ± 0.717 ^a^
Weight reduction rate of larvae (%)	—	21.83 ± 41.57 ^b^	34.85 ± 24.24 ^b^	52.86 ± 18.28 ^a^

Different letters represent significant differences (*p* < 0.05) determined using Dunn’s multiple comparison test (N = 30). The symbol “—” indicates that there is no values here.

## Data Availability

The data presented in this study are openly available in NCBI, reference number [CP114033-CP114034].
